# Real-world experience with direct-acting antiviral therapy in HCV-infected patients with cirrhosis and esophageal varices

**DOI:** 10.1007/s43440-024-00639-9

**Published:** 2024-08-20

**Authors:** Michał Brzdęk, Dorota Zarębska-Michaluk, Michał Kukla, Justyna Janocha-Litwin, Dorota Dybowska, Ewa Janczewska, Beata Lorenc, Hanna Berak, Włodzimierz Mazur, Magdalena Tudrujek-Zdunek, Jakub Klapaczyński, Anna Piekarska, Marek Sitko, Łukasz Laurans, Anna Parfieniuk-Kowerda, Robert Flisiak

**Affiliations:** 1 Collegium Medicum, Jan Kochanowski University, aleja IX Wieków Kielc 19A, Kielce, 25-317 Poland; 2 Department of Infectious Diseases and Allergology, Jan Kochanowski University, Kielce, 25- 317 Poland; 3 Department of Internal Medicine and Geriatrics, Faculty of Medicine, Jagiellonian University Medical College, Kraków, 31-688 Poland; 4 Department of Endoscopy, University Hospital, Kraków, 30-688 Poland; 5 Department of Infectious Diseases and Hepatology, Wrocław Medical University, Wrocław, 50- 367 Poland; 6 Department of Infectious Diseases and Hepatology, Faculty of Medicine, Collegium Medicum Bydgoszcz, Nicolaus Copernicus University, Toruń, 87-100 Poland; 7 Voivodeship Infectious Observation Hospital in Bydgoszcz, Bydgoszcz, 85-030 Poland; 8 Department of Basic Medical Sciences, School of Public Health in Bytom, Medical University of Silesia, Katowice, 40-055 Poland; 9 Pomeranian Center of Infectious Diseases, Medical University, Gdańsk, 80-214 Poland; 10 Outpatient Clinic, Hospital for Infectious Diseases in Warsaw, Warsaw, 01-201 Poland; 11 Clinical Department of Infectious Diseases in Chorzów, Medical University of Silesia, Katowice, 40-055 Poland; 12 Department of Infectious Diseases, Medical University of Lublin, Lublin, 20-059 Poland; 13 Department of Internal Medicine and Hepatology, The National Institute of Medicine of the Ministry of Interior and Administration, Warszawa, 02-507 Poland; 14 Department of Infectious Diseases and Hepatology, Medical University of Łódź, Łódź, 90- 419 Poland; 15 Department of Infectious and Tropical Diseases, Jagiellonian University, Kraków, 31- 088 Poland; 16 Department of Infectious Diseases, Hepatology and Liver Transplantation, Pomeranian Medical University, Szczecin, 70-204 Poland; 17 Multidisciplinary Regional Hospital in Gorzów Wielkopolski, Gorzów Wielkopolski, 66-400 Poland; 18 Department of Infectious Diseases and Hepatology, Medical University of Białystok, Białystok, 15-089 Poland

**Keywords:** Chronic hepatitis C, Hepatitis C virus, Direct-acting antivirals, Portal hypertension, Esophageal varices, Sustained virologic response

## Abstract

**Background:**

Hepatitis C virus (HCV) infection affects 50 million people worldwide with around 242,000 deaths annually, mainly due to complications such as cirrhosis and hepatocellular carcinoma (HCC). Portal hypertension (PH) caused by cirrhosis leads to severe consequences, including esophageal varices (EV). This study aimed to evaluate the effectiveness and safety of direct-acting antiviral (DAA) treatment in patients with and without EV.

**Methods:**

This retrospective analysis involved consecutive HCV-infected adults undergoing DAA therapy at 22 Polish hepatology centers from July 1, 2015, to December 31, 2022. Patients with cirrhosis were categorized based on the presence of EV diagnosed by gastroscopy. Treatment effectiveness was measured by sustained virologic response (SVR), with safety outcomes monitored for 12 weeks post-treatment.

**Results:**

A population of 3393 HCV-infected patients with cirrhosis was divided into groups with (A, *n* = 976) and without (B, *n* = 2417) EV. Group A showed a significantly higher prevalence of comorbidities and concomitant medications. Genotype (GT)1b infections predominated in both groups, and GT3 infections were more common in the EV group. Group A exhibited more severe liver disease, and higher rates of decompensation, HCC, and HBV co-infection. SVR was significantly higher in group B (91.5% vs. 96.3%, *p* < 0.0001). Male gender, GT3, EV presence, and Child-Pugh grade B were identified as independent negative SVR predictors. Group A had a worse safety profile, with notably higher adverse event incidence and mortality.

**Conclusions:**

DAA therapies are highly effective and well tolerated in patients with cirrhosis, but EV presence predicts poorer virologic responses.

## Introduction

Hepatitis C virus (HCV), a member of the *Flaviviridae* family, is characterized by its small, enveloped virions containing single-stranded positive-sense ribonucleic acid (RNA) [1]. HCV forms a formidable global health challenge and affects approximately 50 million individuals worldwide. This infection annually claims the lives of about 242,000 individuals, with the most severe complications manifesting as cirrhosis and hepatocellular carcinoma (HCC) [2, 3].

In liver cirrhosis, increased resistance within the liver’s blood vessels raises portal pressure, subsequently causing portal hypertension (PH). This condition extends its impact to blood vessels outside the liver, leading to the formation of collateral vessels and arterial widening in the splanchnic and systemic circulations. Consequently, ascites and endoscopic changes such as esophageal varices (EV), gastric varices, splenomegaly, and portal hypertensive gastropathy may develop [4, 5].

The assessment of hepatic venous pressure gradient (HVPG) serves as the “gold standard” for diagnosing PH [6]. A measurement greater than or equal to 10 mmHg indicates the high probability of the presence of varices, while a measurement greater than or equal to 12 mmHg signifies the risk of variceal bleeding [7].

The presence of PH significantly increases the likelihood of a worse outcome in individuals with cirrhosis [8]. Individuals diagnosed with compensated cirrhosis and EV exhibit a 5-year mortality rate of 10%, contrasting with the 1.5% observed in those with compensated cirrhosis but without varices [9]. It is crucial to note up to 50% of patients with cirrhosis, the presence of gastroesophageal varices is observed, and the probability of encountering varices rises with the increasing severity of liver disease [10, 11].

In the era of interferon (IFN)-based antiviral regimens, treatment outcomes were significantly poorer among patients with clinically significant PH (CSPH); than among patients without CSPH [12]. The advent of direct-acting antiviral agents (DAAs) has revolutionized HCV treatment, initially used in combination with IFN such as telaprevir and boceprevir, and later as stand-alone therapies, increasing effectiveness even in patients with liver cirrhosis although it should be noted that those with decompensated cirrhosis achieved lower efficacy [13]. However, despite successful HCV eradication and subsequent regression of liver fibrosis, PH may persist in individuals with advanced liver disease [14]. Research findings indicate that individuals with CSPH remain susceptible to liver decompensation for up to five years after completing antiviral treatment [15]. Moreover, the risk of developing HCC persists in patients with PH and advanced fibrosis, despite successful HCV eradication [16].

Although significant progress has been made, data involving the effectiveness of HCV treatments in patients with PH, derived both from clinical trials and real-world evidence (RWE), are scarce. Therefore, this study aims to fill this knowledge gap by comprehensively assessing and summarizing the effectiveness and safety of HCV treatments in patients with liver cirrhosis and with PH manifested by EV. Additionally, we seek to identify potential factors that may influence treatment responses, emphasizing the importance of further research in optimizing therapeutic outcomes for this patient population.

## Materials and methods

### Study population

The study population was selected from 16,767 adult patients infected with HCV who initiated antiviral treatment with IFN-free regimens from 22 Polish hepatology centers between July 1, 2015, and December 31, 2022. The analysis is part of the EpiTer-2 database, a multicenter observational project evaluating antiviral therapy of HCV-infected patients in routine clinical practice, supported by the Polish Association of Epidemiologists and Infectiologists.

**Fig. 1:**
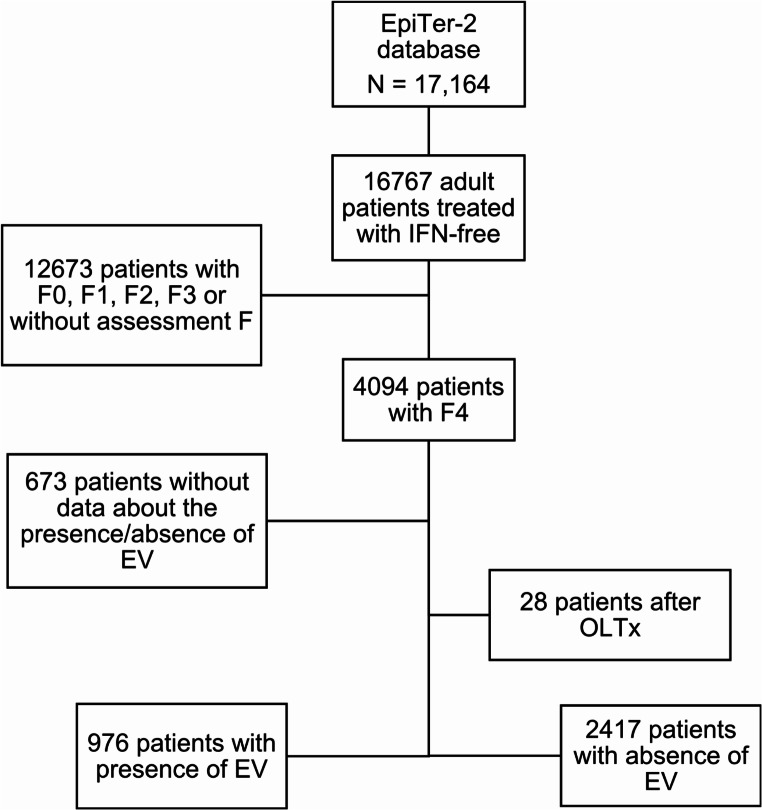
Flow chart of patient selection. Abbreviations: EV, esophageal varices; F, fibrosis; IFN, interferon; OLTx, orthotopic liver transplantation. * The study is a part of the EpiTer-2 database supported by the Polish Association of Epidemiologists and Infectiologists and included adult HCV-infected patients who started IFN-free antiviral treatment at 22 Polish hepatology centers between July 1, 2015, and December 31, 2022

The present study included all consecutive patients with liver cirrhosis from the EpiTer-2 database, who were divided into two groups based on the presence or absence of EV diagnosed through gastroscopy. Patients with a history of liver transplantation and patients who had not undergone gastroscopy were excluded from the analysis (Fig. 1).

The treating physician made treatment decisions, determining the regimen, dosage, and therapy duration. These decisions were guided by the product characteristics, the National Health Fund (NHF) therapeutic program protocol, and recommendations from the Polish Group of Experts for HCV [17–19].

### Data collection

The data were gathered retrospectively through an online questionnaire administered by Tiba LLC based on the medical records.

Parameters collected at the beginning of the study encompassed demographic and clinical data: gender, age, body mass index (BMI), HCV genotype (GT), comorbidities, concomitant medications, information on the severity of the liver disease, coinfections of the human immunodeficiency virus (HIV) and hepatitis B virus (HBV), and the history of previous antiviral therapy. Baseline laboratory parameters including serum alanine transaminase activity, bilirubin, albumin, creatinine concentrations, hemoglobin level, platelet count, as well as international normalized ratio (INR) and HCV viral load, were also recorded. The patient groups were also compared regarding the treatment regimens used and their effectiveness and safety outcomes.

### The severity of liver disease

The advancement of liver disease was assessed by measuring liver stiffness through real-time shear wave elastography using an Aixplorer (SuperSonic Imagine, Aix-en-Provence, France) or transient elastography with FibroScan (Echosens, France). Following the METAVIR score criteria in accordance with the guidelines from the European Association for the Study of the Liver, a cutoff value of 13 kPa was employed to identify individuals with F4, indicating cirrhosis [20]. Patient evaluations included scoring based on Child-Pugh (CP) and the model for end-stage liver disease (MELD).

Additionally, data regarding the presence of EV documented in the endoscopy performed as a part of the qualification for DAA therapy, up to a maximum of 6 months prior to its initiation,

past or present hepatic decompensation, and the history of HCC were collected. Patients scoring as B or C on the CP scale were categorized as decompensated.

### Assessment of treatment effectiveness

A measure of the effectiveness of DAA therapy was sustained virological response (SVR), defined as an undetectable HCV RNA at least 12 weeks after the end of therapy. Patients who then still had a detectable viral load were categorized as virologic nonresponders, whereas those without HCV RNA assessment 12 weeks after the end of therapy due to loss to follow-up were considered non-virological failures. The lower detection limit of all methods used in the analysis through the entire duration of the project was not higher than 15 IU/ml in accordance with national recommendations [17–19].

### Assessment of treatment safety

Safety outcomes were documented throughout the treatment and tracked for 12 weeks after the end of treatment. Throughout the treatment and subsequent follow-up period, the following data were collected: any modification or discontinuation in the treatment plan, the occurrence of adverse events (AE), severe adverse events (SAE), and instances of death along with an assessment of their relationship with antiviral therapy. Adverse events specifically related to liver function, including gastrointestinal bleeding, ascites, and encephalopathy, were closely monitored in patients.

### Ethics

Patient data were collected and analyzed in compliance with relevant data protection regulations [21]. Patients gave their consent to participate in the therapeutic program in accordance with the regulations of the NHF. The initially collected data were not intended for scientific purposes but rather for evaluating the effectiveness and safety of registered medications in real-world clinical settings. Patients did not undergo any experimental treatments. As per the local law applicable during the study (the Polish Pharmaceutical Law of 6 September 2001, art. 37al), non-interventional studies did not necessitate approval from an ethics committee.

### Statistical analysis

Categorical data were presented in the form of numerical values and percentages, and comparisons between groups were made using either Pearson’s χ2 test or the Fisher’s exact test, depending on their suitability. For continuous data, such as age, BMI, and laboratory markers, the median, first quartile (Q) and third Q were used for summarization due to their non-Gaussian distribution, which was confirmed by the Shapiro–Wilk test. Group differences were evaluated through the nonparametric Mann–Whitney test. For multiple comparisons, the Bonferroni correction was applied. The SVR was assessed for all patients who initiated treatment, after excluding those lost to follow-up in accordance with the per-protocol (PP) analysis. Multiple logistic regression was used to predict the odds of no response to HCV treatment. *P* values of < 0.05 were considered to be statistically significant. Statistical analyses were performed using Statistica v. 13 (StatSoft, Tulsa, OK, United States) and GraphPad Prism 5.1 (GraphPad Software, Inc., La Jolla, CA).

## Results

### Patient characteristics

The analyzed population consisted of 3393 individuals with cirrhosis and chronic HCV infection who underwent IFN-free regimens. The population was categorized according to their EV status: one group comprising patients with EV (*n* = 976; 28.8%; group A) and the other consisting of those without EV (*n* = 2417; 71.2%; group B). In both analyzed groups, a male predominance was observed, and there were no differences in the median of age between the two groups. Patients in group B exhibited a statistically significantly higher BMI compared to patients in group A (*p* = 0.0004, Mann–Whitney test) (Table 1).

**Table 1:** The comparison of baseline characteristics of patients with esophageal varices and without esophageal varices*

Baseline characteristics	Patients with EV (*n* = 976)	Patients without EV (*n* = 2417)	*p*-value
Gender, females/males, n (%)/ n (%)	408 (41.8)/568 (58.2)	1085 (44.9)/1332 (55.1)	0.1711^1^
Age [years], median (Q1-Q3)	58.0 (50.0–65.0)	59.0 (49.0–67.0)	0.5631^2^
Females [years], median (Q1-Q3)	62.0 (56.0–69.0)	62.0 (55.0–69.0)	0.9861^2^
Males [years], median (Q1-Q3)	56.0 (46.0–62.0)	55.0 (44.0–63.0)	0.8590^2^
BMI median (Q1-Q3)	26.7 (23.8–29.4)	27.1 (24.4–30.3)	0.0004^2^
Comorbidities, n (%)			
Any comorbidity, n (%)	778 (79.7)	1804 (74.6)	0.0119^1^
Hypertension, n (%)	401 (41.1)	1149 (47.5)	0.0045^1^
Diabetes, n (%)	237 (24.3)	533 (22.1)	1^1^
Renal disease, n (%)	54 (5.5)	85 (3.5)	0.0512^1^
Autoimmune diseases, n (%)	22 (2.3)	42 (1.7)	1^1^
Non-HCC tumors, n (%)	23 (2.4)	59 (2.4)	1^1^
Other, n (%)	612 (62.7)	1245 (51.5)	< 0.0001^1^
Concomitant medications, n (%)	858 (87.9)	1672 (69.2)	< 0.0001^1^
Beta-Blockers, n (%)	524 (53.7)	640 (26.5)	< 0.0001^1^
Rifaximin, n (%)	30 (3.1)	6 (0.2)	< 0.0001^1^
ALT IU/L, median (Q1-Q3)	71.0 (46.0-111.0)	82.6 (52.0-133.0)	< 0.0001^2^
Bilirubin mg/dL, median (Q1-Q3)	1.2 (0.9–1.8)	0.8 (0.6–1.2)	< 0.0001^2^
Albumin g/dL, median (Q1-Q3)	3.6 (3.2–3.9)	4.0 (3.7–4.3)	< 0.0001^2^
INR, median (Q1-Q3)	1.2 (1.1–1.3)	1.1 (1.0-1.2)	< 0.0001^2^
Creatinine mg/dL, median (Q1-Q3)	0.8 (0.7–0.9)	0.8 (0.7–0.9)	0.1089^2^
Hemoglobin g/dL, median (Q1-Q3)	13.6 (12.3–14.7)	14.4 (13.2–15.4)	< 0.0001^2^
Platelets, x1000/µL, median (Q1-Q3)	85.0 (63.0-117.0)	136.0 (97.0-183.5)	< 0.0001^2^
HCV RNA x10^6^ IU/mL, median (Q1-Q3)	0.5 (0.2–1.4)	1.0 (0.3–2.6)	< 0.0001^2^

^1^ Data were analysed using Pearson’s χ2 test

^2^ Data were analysed using Mann–Whitney test

The Bonferroni correction was applied to account for multiple comparisons. *P* values of < 0.05 were considered to be statistically significant

Abbreviations: ALT, alanine transaminase; BMI, body mass index; EV, esophageal varices; HBc, hepatitis B core; HBsAg, hepatitis B surface; HBV, hepatitis B virus; HCC, hepatocellular carcinoma; HCV, hepatitis C virus; INR, international normalized ratio; Q, quartile; RNA, ribonucleic acid

* The study is a part of the EpiTer-2 database supported by the Polish Association of Epidemiologists and Infectiologists and included adult HCV-infected patients who started IFN-free antiviral treatment at 22 Polish hepatology centers between July 1, 2015, and December 31, 2022. Patients with cirrhosis were divided based on the presence (*n* = 976) or absence of esophageal varices (*n* = 2417) as determined by gastroscopy. The table presents the number and percentage [n (%)] for qualitative variables and the median (Q1-Q3), which includes the median, first quartile, and third quartile, for quantitative variables in both analyzed groups

Patients in group A had a significantly higher prevalence of comorbidities (*p* = 0.0119, Pearson’s χ2 test); however, hypertension, the most common coexisting disease, occurred significantly more frequently in group B (*p* = 0.0045, Pearson’s χ2 test). The rate of patients using concomitant medications was significantly higher among those in group A (*p* < 0.0001, Pearson’s χ2 test).

Individuals with EV exhibited significantly lower albumin levels (*p* < 0.0001, Mann–Whitney test), hemoglobin concentration (*p* < 0.0001, Mann–Whitney test), platelet count (*p* < 0.0001, Mann–Whitney test), baseline viral load (*p* < 0.0001, Mann–Whitney test), and significantly higher values of INR (*p* < 0.0001, Mann–Whitney test), as well as bilirubin concentration (*p* < 0.0001, Mann–Whitney test).

### Characteristics of HCV infection and liver disease severity

GT1b was the dominant genotype in both groups; however, infections of GT3 were significantly more prevalent in groups with EV (*p* = 0.0271, Pearson’s χ2 test). Among patients with EV, the prevalence of individuals classified at baseline as B or C in the CP scale was significantly higher (*p* < 0.0001, Pearson’s χ2 test) (Table 2). Patients without EV were more likely to be rated as grade A and a slightly greater proportion of patients had a MELD score below 15 than those in group A (*p* < 0.0001, Pearson’s χ2 test). A statistically significant higher rate of decompensation in the past (*p* < 0.0001, Pearson’s χ2 test) and at baseline (*p* < 0.0001, Pearson’s χ2 test) was observed among patients in group A compared to group B. In addition, the prevalence of HCC (*p* = 0.0001, Pearson’s χ2 test) and co-infection with HBV (*p* = 0.0059, Pearson’s χ2 test) was found to be higher in group A. Whereas, the incidence of HIV was observed to be similar in both groups.

**Table 2:** The comparison of characteristics of HCV infection and liver disease in patients with esophageal varices and without esophageal varices*

Characteristics of HCV infection and liver disease	Patients with EV (*n* = 976)	Patients without EV (*n* = 2417)	*p*-value
GT, n (%)			0.1001^1^
GT1, n (%)	19 (2.0)	49 (2.0)	
GT1a, n (%)	20 (2.0)	55 (2.3)	
GT1b, n (%)	742 (76.0)	1890 (78.2)	
GT2, n (%)	0	8 (0.3)	
GT3, n (%)	166 (17.0)	339 (14.0)	
GT4, n (%)	28 (2.9)	76 (3.2)	
GT6, n (%)	1 (0.1)	0	
GT3/nonGT3, n (%)/n (%)	166 (17.0)/810 (83.0)	339 (14.0)/2078 (86.0)	0.0271^1^
GT1b/nonGT1b, n (%)/n (%)	742 (76.0)/234 (24)	1890 (78.2)/527 (21.8)	0.1698^1^
CP, n (%)			< 0.0001^1^
A, n (%)	730 (74.8)	2277 (94.2)	
B, n (%)	230 (23.6)	134 (5.5)	
C, n (%)	16 (1.6)	6 (0.3)	
MELD score, median (Q1-Q3)	10 (8–12)	8 (7–9)	< 0.0001^2^
MELD score, n (%)			0.0001^1^
< 15, n (%)	875 (89.7)	2234 (92.4)	
15–18, n (%)	60 (6.1)	70 (2.9)	
19–20, n (%)	10 (1.0)	27 (1.1)	
> 20, n (%)	6 (0.6)	24 (1.0)	
No data, n (%)	25 (2.6)	62 (2.6)	
History of hepatic decompensation, n (%)			
Ascites, n (%)	280 (28.7)	100 (4.1)	< 0.0001^1^
Encephalopathy, n (%)	70 (7.2)	23 (1.0)	< 0.0001^1^
Hepatic decompensation at baseline, n (%)			
Moderate ascites– responded to diuretics, n (%)	135 (13.8)	58 (2.4)	< 0.0001^1^
Tense ascites– not responded to diuretics, n (%)	10 (1.0)	2 (0.1)	0.0001^3^
Encephalopathy, n (%)	57 (5.8)	23 (1.0)	< 0.0001^1^
HCC history, n (%)	62 (6)	81 (3.4)	0.0001^1^
HBV coinfection (HBsAg+), n (%)	22 (2.3)	25 (1.0)	0.0059^1^
HIV coinfection, n (%)	20 (2.0)	59 (2.4)	0.4932^1^

^1^ Data were analysed using Pearson’s χ2 test

^2^ Data were analysed using Mann–Whitney test

^3^ Data were analysed using Fisher’s exact test

The Bonferroni correction was applied to account for multiple comparisons. *P* values of < 0.05 were considered to be statistically significant

Abbreviations: CP, Child-Pugh; EV, esophageal varices; GT, genotype, HCV, hepatitis C virus; HBsAg, hepatitis B surface antigen; HBV, hepatitis B virus; HCC, hepatocellular carcinoma; HIV, human immunodeficiency virus; Q, quartile; MELD, a model for end-stage liver disease

* The study is a part of the EpiTer-2 database supported by the Polish Association of Epidemiologists and Infectiologists and included adult HCV-infected patients who started IFN-free antiviral treatment at 22 Polish hepatology centers between July 1, 2015, and December 31, 2022. Patients with cirrhosis were divided based on the presence (*n* = 976) or absence of esophageal varices (*n* = 2417) as determined by gastroscopy. The table presents the number and percentage [n (%)] for qualitative variables and the median (Q1-Q3), which includes the median, first quartile, and third quartile, for quantitative variables in both analyzed groups

### Treatment characteristics and effectiveness

The percentage of treatment-naive patients was higher in group B than in group A, and the difference was statistically significant (*p* = 0.0026, Pearson’s χ2 test) (Table 3). The majority of patients in both groups received genotype-specific regimens, and this prevalence was higher in the population with EV (*p* = 0.0009, Pearson’s χ2 test). In the group of patients with EV, the addition of ribavirin (RBV) was significantly more frequently utilized, especially in patients with GT3, compared to those without EV (*p* < 0.0001, Pearson’s χ2 test).

**Table 3:** The comparison of antiviral treatment characteristics in patients with esophageal varices and without esophageal varices*

Antiviral treatment characteristics	Patients with EV (*n* = 976)	Patients without EV (*n* = 2417)	*p*-value
History of previous therapy, n (%)			0.0026^1^
Treatment-naïve, n (%)	650 (66.6)	1765 (73.0)	
Null-responder, n (%)	138 (14.2)	249 (10.3)	
Relapser, n (%)	99 (10.1)	213 (8.8)	
Discontinuation due to safety reasons, n (%)	41 (4.2)	78 (3.2)	
Unknown type of response, n (%)	48 (4.9)	112 (4.7)	
Current treatment regimen, n (%)			
Genotype-specific treatment regimens, n (%)	622 (63.7)	1391 (57.6)	0.0009^1^
ASV + DCV, n (%)	16 (1.6)	30 (1.2)	1^1^
LDV/SOF ± RBV, n (%)	302 (30.9)	424 (17.5)	< 0.0001^1^
OBV/PTV/r ± DSV ± RBV, n (%)	237 (24.3)	649 (26.9)	1^1^
GZR/ EBR ± RBV, n (%)	67 (6.9)	288 (11.9)	0.0001^1^
Pangenotypic regimens, n (%)	354 (36.3)	1026 (42.4)	0.0099^1^
GLE/PIB, n (%)	81 (8.3)	416 (17.2)	< 0.0001^1^
GLE/PIB + SOF + RBV, n (%)	1 (0.1)	1 (0.1)	1^1^
SOF/VEL ± RBV, n (%)	194 (19.9)	527 (21.8)	1^1^
SOF/VEL/VOX, n (%)	6 (0.6)	11 (0.5)	1^1^
SOF + RBV, n (%)	65 (6.7)	56 (2.3)	< 0.0001^1^
SOF + SMV ± RBV, n (%)	1 (0.1)	2 (0.1)	> 0.9999^2^
SOF + DCV ± RBV, n (%)	6 (0.6)	13 (0.5)	1^1^
Current-RBV-containing therapies, n (%)	492 (50.4)	779 (32.2)	< 0.0001^1^

^1^ Data were analysed using Pearson’s χ2 test

^2^ Data were analysed using Fisher’s exact test

*P* values of < 0.05 were considered to be statistically significant

Abbreviations: ASV, asunaprevir; DCV, daclatasvir; DSV, dasabuvir; EBR, elbasvir; EV, esophageal varices; GLE, glecaprevir; GT, genotype; GZR, grazoprevir; LDV, ledipasvir; OBV, ombitasvir; PIB, pibrentasvir; PTV/r, paritaprevir boosted with ritonavir; RBV, ribavirin; SMV, simeprevir; SOF, sofosbuvir; VEL, velpatasvir; VOX, voxilaprevir

* The study is a part of the EpiTer-2 database supported by the Polish Association of Epidemiologists and Infectiologists and included adult HCV-infected patients who started IFN-free antiviral treatment at 22 Polish hepatology centers between July 1, 2015, and December 31, 2022. Patients with cirrhosis were divided based on the presence (*n* = 976) or absence of esophageal varices (*n* = 2417) as determined by gastroscopy. The table presents the number and percentage [n (%)] for qualitative variables in both analyzed groups

**Fig. 2:**
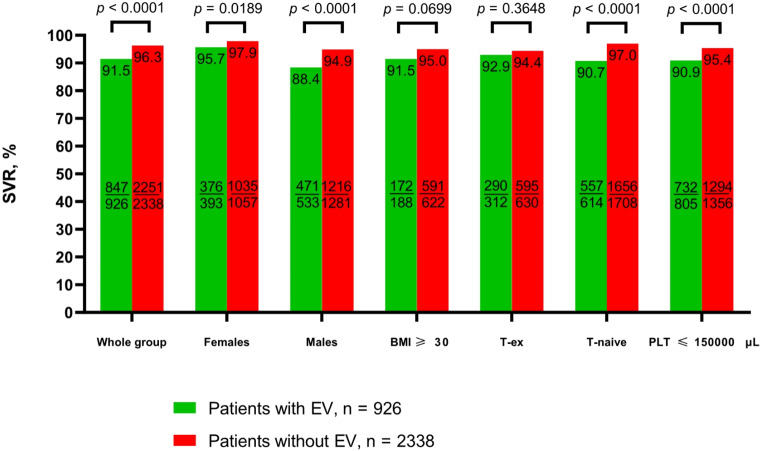
Comparison of SVR in a population of patients with and without esophageal varices, taking into account gender, BMI, history of previous therapy, and platelet count. Data were analyzed using Pearson’s χ2 test. *P* values of < 0.05 were considered to be statistically significant. Abbreviations: BMI, body mass index, PP, per protocol; PLT, platelet count; T, treatment; SVR, sustained virologic response. * The study is a part of the EpiTer-2 database supported by the Polish Association of Epidemiologists and Infectiologists and included adult HCV-infected patients who started IFN-free antiviral treatment at 22 Polish hepatology centers between July 1, 2015, and December 31, 2022. The bars in the figure present the sustained virologic response rate in patients with and without esophageal varices according to gender, BMI, history of previous therapy, and platelet count. Detailed rates are shown on the tops of the bars. Additionally, within the bars, the number of patients with SVR relative to the total number in each subgroup is displayed. Patients lost to follow-up were excluded from the analysis (per-protocol analysis)

**Fig. 3:**
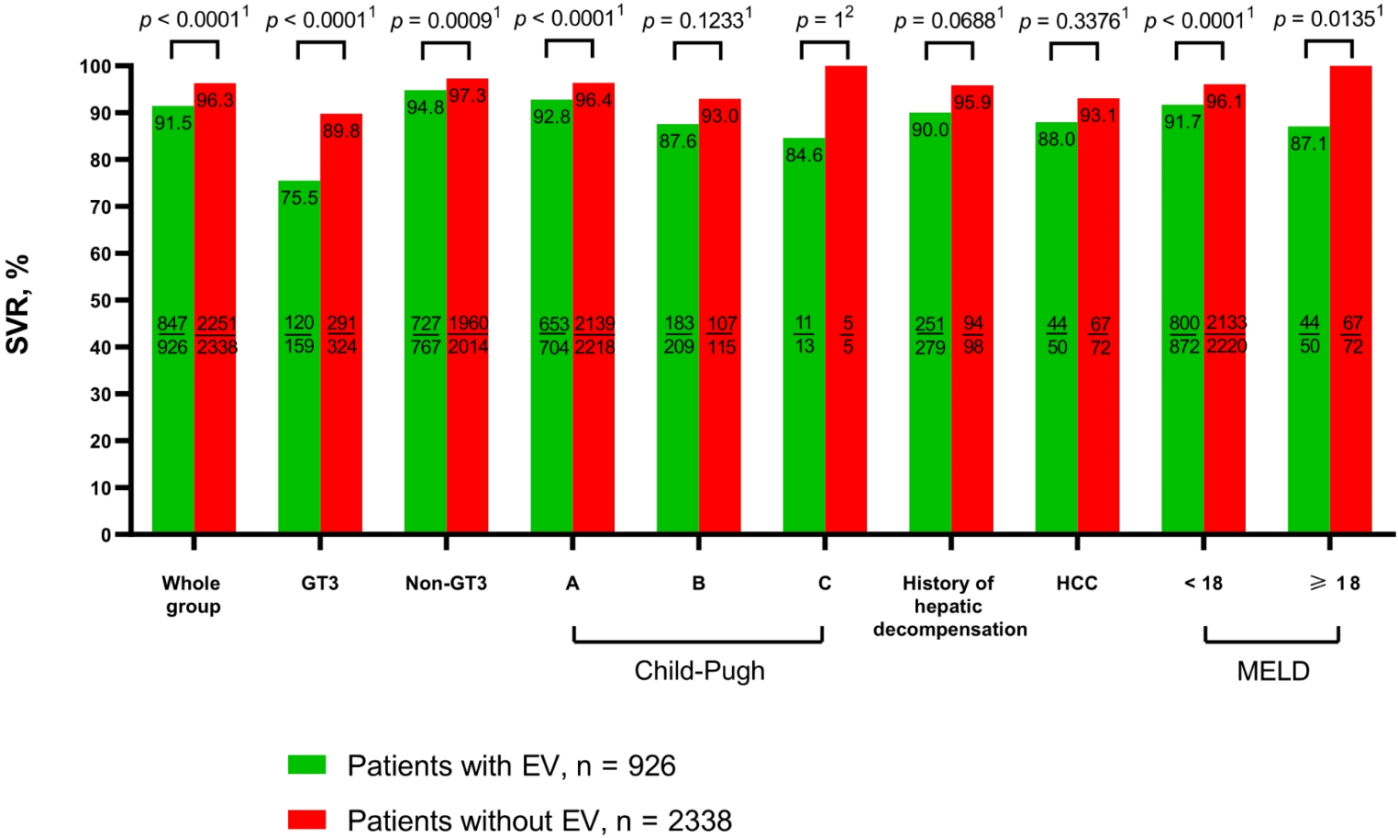
Comparison of SVR in a population of patients with and without esophageal varices with consideration of HCV genotype, Child-Pugh score and MELD, history of liver function decompensation, and HCC. ^1^ Data were analysed using Pearson’s χ2 test. ^2^ Data were analysed using Fisher’s exact test. *P* values of < 0.05 were considered to be statistically significant. Abbreviations: CP, Child-Pugh; GT, genotype; HCC, hepatocellular carcinoma; MELD, model for end-stage liver disease; PP, per protocol; SVR, sustained virologic response. *The study is a part of the EpiTer-2 database supported by the Polish Association of Epidemiologists and Infectiologists and included adult HCV-infected patients who started IFN-free antiviral treatment at 22 Polish hepatology centers between July 1, 2015, and December 31, 2022. The bars in the figure present the sustained virologic response in patients with esophageal varices and without esophageal varices as well as according to GT, CP, history of hepatic decompensation, history of HCC, and MELD. Detailed rates are shown on the tops of the bars. Additionally, within the bars, the number of patients with SVR relative to the total number in each subgroup is displayed. Patients lost to follow-up were excluded from the analysis (per-protocol analysis)

A total of 96.2% of patients had SVR data available; the effectiveness of therapy calculated in this population was significantly lower in patients with EV compared to those without EV (*p* < 0.0001, Pearson’s χ2 test) (Fig. 2). This difference was observed in both genders. In treatment-naïve patients, a significantly worse response to therapy was noted in group A (*p* < 0.0001, Pearson’s χ2 test). The lowest effectiveness of therapy was documented in the GT3-infected population, with an SVR of 75.5% in the EV group and 89.8% in patients without EV, and the difference was statistically significant (*p* < 0.0001, Pearson’s χ2 test*)* (Fig. 3). There was no statistically significant difference in SVR according to the history of liver decompensation; however, it was noted in subgroups based on CP and MELD scores at the start of antiviral therapy. Patients with EV and MELD scores < 15 and 15–18 had significantly lower effectiveness than those without EV, (*p* < 0.0001, Pearson’s χ2 test) and (*p* = 0.0423, Pearson’s χ2 test), respectively. Patients with MELD scores of 19–20 and > 20 achieved higher SVR rates in the EV group compared to patients without EV, but the difference was not statistically significant.

Among patients with GT3 infection and EV treated with new pangenotypic options (glecaprevir (GLE) / pibrentasvir (PIB), sofosbuvir (SOF) / velpatasvir (VEL) ± RBV, GLE/PIB + SOF + RBV, SOF/VEL/ voxilaprevir (VOX)), an SVR rate was significantly lower than in the group of patients without EV (*p* = 0.0009, Pearson’s χ2 test). Comparative analysis of the effectiveness of the different regimens regardless of the GT showed a significantly worse response in the EV patient population for the GLE/PIB (*p =* 0.0008, Pearson’s χ2 test) and SOF/VEL ± RBV (*p* = 0.0019, Pearson’s χ2 test), while the other regimens resulted in a comparable SVR; detailed results are presented in Table 4.

**Table 4:** The comparison of SVR according to the treatment regimen*

Treatment regimen	Patients with EV, *n*/*N* (%)	Patients without EV, *n*/*N* (%)	*p*-value
LDV/SOF ± RBV, n/N (%)	271/286 (94.8)	393/408 (96.3)	0.3173
OBV/PTV/r ± DSV ± RBV, n/N (%)	219/227 (96.5)	623/634 (98.3)	0.1153
GLE/PIB, n/N (%)	69/77 (89.6)	393/403 (97.5)	0.0008
SOF/VEL ± RBV, n/N (%)	156/181 (86.2)	471/503 (93.6)	0.0019
SOF + RBV, n/N (%)	43/61 (70.5)	43/52 (82.7)	0.1296

Data were analysed using Pearson’s χ2 test

P values of < 0.05 were considered to be statistically significant

Abbreviations: EV, esophageal varices; GLE, glecaprevir; LDV, ledipasvir; OBV, ombitasvir; PIB, pibrentasvir; PTV/r, paritaprevir boosted with ritonavir; RBV, ribavirin; SOF, sofosbuvir; VEL, velpatasvir

* The study is a part of the EpiTer-2 database supported by the Polish Association of Epidemiologists and Infectiologists and included adult HCV-infected patients who started IFN-free antiviral treatment at 22 Polish hepatology centers between July 1, 2015, and December 31, 2022. The table presents the number of patients with SVR (n) treated with following regimens relative to the total number of patients treated with each regimen (N), as well as the percentage (%) in both analyzed groups. Patients lost to follow-up were excluded from the analysis (per-protocol analysis)

Virologic nonresponders were more likely to be males (*p* < 0.0001, Pearson’s χ2 test), had a significantly younger age (*p* = 0.0001, Mann–Whitney test), and higher BMI (*p* = 0.0008, Mann–Whitney test), as well as were significantly more likely to be infected with GT3 (*p* < 0.0001, Pearson’s χ2 test) (Table 5). Among them, decompensation of liver function in the form of encephalopathy (*p* = 0.0129, Pearson’s χ2 test) or moderate ascites at baseline was documented significantly more often (*p* = 0. 0001, Pearson’s χ2 test), as well as CP score of B or C (*p* < 0.0001, Pearson’s χ2 test), presence of EV (*p* < 0.0001, Pearson’s χ2 test) and history of ascites (*p* = 0.0039, Pearson’s χ2 test). Those who did not respond virologically to treatment more frequently received the pangenotypic options compared to successfully treated patients (*p* < 0.0001, Pearson’s χ2 test).

**Table 5:** The comparison of virological responders and nonresponders to DAA therapy*

Characteristics	Responders (*n* = 3098)	Nonresponders (*n* = 166)	*p*-value
Gender, females/males, *n* (%)/ *n* (%)	1411 (45.5)/1687 (54.5)	39 (23.5)/127 (76.5)	< 0.0001^1^
Age (yr), median (Q1-Q3)	59.0 (49.0–66.0)	54.0 (48.0–62.0)	0.0001^2^
BMI, median (Q1-Q3)	26.9 (24.2–30.0)	27.8 (25.9–30.8)	0.0008^2^
Current treatment regimen, n (%)			
Genotype-specific treatment regimens, n (%)	1884 (60.8)	63 (38.0)	< 0.0001^1^
ASV + DCV, n (%)	41 (1.3)	5 (3.0)	0.7936^3^
LDV/SOF ± RBV, n (%)	664 (21.4)	30 (18.1)	1^1^
OBV/PTV/r ± DSV ± RBV, n (%)	842 (27.2)	19 (11.4)	0.0001^1^
GZR/EBR ± RBV, n (%)	337 (10.9)	9 (5.4)	0.2871^1^
Pangenotypic regimens, n (%)	1214 (39.2)	103 (62.0)	< 0.0001^1^
GLE/PIB, n (%)	462 (14.9)	18 (10.8)	1^1^
GLE/PIB + SOF + RBV, n (%)	2 (0.1)	0	1^3^
SOF/VEL ± RBV, n (%)	627 (20.2)	57 (34.3)	0.0001^1^
SOF/VEL/VOX, n (%)	15 (0.5)	1 (0.6)	1^3^
SOF + RBV, n (%)	86 (2.8)	27 (16.4)	< 0.0001^1^
SOF + SMV ± RBV, n (%)	3 (0.1)	0	1^3^
SOF + DCV ± RBV, n (%)	19 (0.6)	0	1^3^
Current-RBV-containing therapies, n (%)	1152 (37.2)	81 (48.8)	0.0026^1^
Comorbidities, *n* (%)			
Any, n (%)	2351 (75.9)	130 (78.3)	1^1^
Hypertension, n (%)	1433 (46.3)	65 (39.2)	0.4424^1^
Diabetes, n (%)	697 (22.5)	42 (25.3)	1^1^
Autoimmune diseases, n (%)	58 (1.9)	4 (2.4)	1
Renal disease, n (%)	122 (3.9)	8 (4.8)	1^1^
Non-HCC tumors, n (%)	69 (2.2)	6 (3.6)	1^1^
Concomitant medications, *n* (%)	2287 (73.8)	139 (83.7)	0.0044^1^
GT, n (%)			
GT3/nonGT3, n (%)/ n (%)	411 (13.3)/2687 (86.7)	72 (43.4)/94 (56.6)	< 0.0001^1^
GT1b/nonGT1b, n (%)/ n (%)	2445 (78.9)/653 (21.1)	90 (54.2)/76 (45.8)	< 0.0001^1^
History of previous therapy, n (%)			0.1099^1^
Treatment-naïve, n (%)	2213 (71.4)	109 (65.7)	
Treatment-experienced, n (%)	885 (28.6)	57 (34.3)	
History of hepatic decompensation, *n* (%)			
Ascites, n (%)	321 (10.4)	29 (17.5)	0.0039^1^
Encephalopathy, n (%)	75 (2.4)	8 (4.8)	0.0558^1^
Documented EV, *n* (%)	847 (27.3)	79 (47.6)	< 0.0001^1^
Hepatic decompensation at baseline, *n* (%)			
Encephalopathy, n (%)	61 (2.0)	8 (4.8)	0.0129^1^
Moderate ascites– responded to diuretics, n (%)	155 (5.0)	20 (12.0)	0.0001^1^
Tense ascites– not responded to diuretics, n (%)	9 (0.3)	0	1^3^
HCC history, *n* (%)	111 (3.6)	11 (6.6)	0.0440^1^
CP, *n* (%)			< 0.0001^1^
A, n (%)	2792 (90.1)	130 (78.3)	
B, n (%)	290 (9.4)	34 (20.5)	
C, n (%)	16 (0.5)	2 (1.2)	
HIV co-infection, n (%)	71 (2.3)	5 (3.0)	0.5488^3^
HBV co-infection (HBsAg+), n(%)	45 (1.5)	2 (1.2)	1^3^

^1^ Data were analysed using Pearson’s χ2 test

^2^ Data were analysed using Mann–Whitney test

^3^ Data were analysed using Fisher’s exact test

The Bonferroni correction was applied to account for multiple comparisons. *P* values of < 0.05 were considered to be statistically significant

Abbreviations: ASV, asunaprevir; BMI, body mass index; CP, Child-Pugh; DAA, direct-acting antiviral; DCV, daclatasvir; DSV, dasabuvir; EBR, elbasvir; EV, esophageal varices; GLE, glecaprevir; GT, genotype; GZR, grazoprevir; HBsAg, hepatitis B surface antigen; HBV, hepatitis B virus; HCC, hepatocellular carcinoma; HIV, human immunodeficiency virus; Q, quartile; LDV, ledipasvir; OBV, ombitasvir; PIB, pibrentasvir; PTV/r, paritaprevir boosted with ritonavir; RBV, ribavirin; SMV, simeprevir; SOF, sofosbuvir; VEL, velpatasvir; VOX, voxilaprevir

* The study is a part of the EpiTer-2 database supported by the Polish Association of Epidemiologists and Infectiologists and included adult HCV-infected patients who started IFN-free antiviral treatment at 22 Polish hepatology centers between July 1, 2015, and December 31, 2022. The table presents the number and percentage [n (%)] for qualitative variables and the median (Q1-Q3), which includes the median, first quartile, and third quartile, for quantitative variables in groups of responders and nonresponders to DAA therapy

Independent negative predictors of SVR in the logistic regression analysis were male gender [odds ratio (OR) = 2.110, *p* = 0.0002], GT3 infection (OR = 4.441, *p* < 0.0001), documented EV (OR = 1.847, *p* = 0.0013), and CP scores of B (OR = 1.781, *p* = 0.0348) while, there was no association with age, BMI, treatment experience, RBV use, history of HCC, encephalopathy at baseline and history of ascites (Table 6).

**Table 6:** Factors associated with virologic failure in the logistic regression model in 3393 analyzed HCV-infected patients*

Characteristics	Effect Measure	Wald stat	OR	95%CI	*p*-value
Intercept		311.106	0.009	0.006–0.016	< 0.0001
Age	≥ 65 years	1.282	1.295	0.828–2.027	0.2574
Gender	Males	13.679	2.110	1.421–3.135	0.0002
BMI	≥ 30 kg/m^2^	1.745	1.284	0.886–1.862	0.1865
GT3	Yes	69.540	4.441	3.128–6.305	< 0.0001
Documented EV	Yes	10.399	1.847	1.272–2.682	0.0013
Treatment experience	Yes	3.107	1.397	0.963–2.026	0.078
Moderate ascites at baseline (responded to diuretics)	Yes	2.342	1.719	0.859–3.438	0.1259
Encephalopathy at baseline	Yes	0.507	1.382	0.567–3.369	0.4765
History of Ascites	Yes	0.125	0.905	0.520–1.576	0.7239
CP	B	4.455	1.781	1.042–3.045	0.0348
CP	C	0.013	1.108	0.191–6.423	0.9088
HCC history	Yes	1.332	1.554	0.735–3.283	0.2484
RBV use	Yes	0.901	1.190	0.831–1.704	0.3426

Abbreviations: BMI, Body Mass Index; CP, Child-Pugh; CI, Confidence interval; GT, genotype; EV, esophageal varices; OR, Odds ratio; HCC, hepatocellular carcinoma, RBV, ribavirin

* The study is a part of the EpiTer-2 database supported by the Polish Association of Epidemiologists and Infectiologists and included adult HCV-infected patients who started IFN-free antiviral treatment at 22 Polish hepatology centers between July 1, 2015, and December 31, 2022. The table presents the results of the logistic regression analysis, which was conducted to determine independent negative predictors of sustained virologic response

### Treatment safety

The majority of patients in both groups completed the planned treatment course. Changes to the treatment plan typically occur in connection with a decrease in dosage or discontinuation of RBV due to anemia. Overall, the safety profile was notably worse in the subpopulation with EV, showing a significantly higher incidence of AEs (*p* < 0.0001, Pearson’s χ2 test), including serious ones (*p* < 0.0001, Pearson’s χ2 test), and those leading to treatment discontinuation (*p* < 0.0001, Pearson’s χ2 test). Adverse events such as worsening ascites, emerging hepatic encephalopathy, and gastrointestinal bleeding were also more prevalent in this population (*p* < 0.0001, Pearson’s χ2 test; *p* < 0.0001, Pearson’s χ2 test and *p* < 0.0001, Pearson’s χ2 test, respectively). Although mortality was significantly higher in group A (*p* < 0.0001, Pearson’s χ2 test), none of the deaths in both groups were reported by the treating physician as related to antiviral treatment (Table 7).

**Table 7:** The comparison of safety of DAA therapy in patients with esophageal varices and without esophageal varices*

Safety parameters	Patients with EV (*n* = 976)	Patients without EV (*n* = 2417)	*p*-value
Treatment course, n (%)			< 0.0001^1^
according to schedule, n (%)	891 (91.3)	2320 (96.0)	
therapy modification, n (%)	48 (4.9)	51 (2.1)	
therapy discontinuation, n (%)	36 (3.7)	41 (1.7)	
No data, n (%)	1 (0.1)	5 (0.2)	
Patients with at least one AE, n (%)	391 (40.1)	545 (22.5)	< 0.0001^1^
Serious adverse events, n (%)	53 (5.4)	41 (1.7)	< 0.0001^1^
AEs leading to treatment discontinuation, n (%)	32 (3.3)	30 (1.2)	< 0.0001^1^
Most common AEs (≥ 2%), *n* (%)			
Weakness/fatigue, n (%)	175 (17.9)	231 (9.6)	< 0.0001^1^
Anemia, n (%)	68 (7.0)	34 (1.4)	< 0.0001^1^
Headaches, n (%)	18 (1.8)	57 (2.4)	0.3566^1^
Itchy skin, n (%)	37 (3.8)	73 (3.0)	0.2512^1^
Sleep disorders, n (%)	30 (3.1)	64 (2.6)	0.4939^1^
Hyperbilirubinemia, n (%)	37 (3.8)	31 (1.3)	< 0.0001^1^
AEs of particular interest, n (%)			
Ascites, n (%)	68 (7.0)	25 (1.0)	< 0.0001^1^
hepatic encephalopathy, n (%)	39 (4.0)	14 (0.6)	< 0.0001^1^
gastrointestinal bleeding, n (%)	15 (1.5)	4 (0.2)	< 0.0001^2^
Death, n (%)	30 (3.1)^3^	24 (1.0)^4^	< 0.0001^1^

*P* values of < 0.05 were considered to be statistically significant

^1^ Data were analysed using Pearson’s χ2 test

^2^ Data were analysed using Fisher’s exact test

^3^Sixteen deaths were related to liver disease (gastrointestinal bleeding, hepatic decompensation, progression of HCC), 3 deaths were not associated with liver disease, no data on the cause of death for 11 patients

^4^Three deaths were related to liver disease (hepatic decompensation, progression of HCC), 8 deaths were not associated with liver disease, and no data on the cause of death for 13 patients

Abbreviations: AE, adverse event; DAA, direct-acting antiviral; EV, esophageal varices

* The study is a part of the EpiTer-2 database supported by the Polish Association of Epidemiologists and Infectiologists and included adult HCV-infected patients who started IFN-free antiviral treatment at 22 Polish hepatology centers between July 1, 2015, and December 31, 2022. Patients with cirrhosis were divided based on the presence (*n* = 976) or absence of esophageal varices (*n* = 2417) as determined by gastroscopy. The table presents the number and percentage [n (%)] for qualitative variables in both analyzed groups

## Discussion

In this multicenter retrospective real-world cohort study, we revealed that among patients infected with HCV and cirrhosis, those exhibiting the presence of EV achieved an SVR rate of 91.5%. In contrast, a higher SVR rate of 96.3% was observed in the subgroup of patients without EV.

During the era of IFN-based antiviral regimens, the effectiveness of IFN-based antiviral therapies was markedly low, particularly in patients with CSPH (HVPG ≥ 10 mm), with a success rate below 20% in patients infected with GT1 [12]. Moreover, the administration of IFN-based therapies was often impeded by the prevalence of hematologic complications, including leukopenia, thrombocytopenia, and anemia, which were attributed to the myelosuppressive effects of IFN [22]. However, portal pressure also played a significant role in exacerbating these complications [23]. The aforementioned hematologic complications, particularly thrombocytopenia and leukopenia, frequently necessitated treatment discontinuation in this patient population [24].

The introduction of DAA marked a breakthrough in treating patients with HCV infection, including those with cirrhosis, significantly improving both the effectiveness and safety profile of the treatment [25]. There is a notable lack of studies comparing the efficacy and safety of IFN-free therapies in patients with and without CSPH [26–29].

A prospective study of Italian patients with GT3 HCV and liver cirrhosis treated with SOF/VEL without RBV for 12 weeks found that the presence of EV did not significantly impact the treatment’s effectiveness [26]. This study revealed similarly high SVR rates of 98.4% in patients without EV and 97.1% in those with varices, moreover in the subgroup of patients deemed at high risk of PH (determined by non-invasive evaluation), the SVR rate was 99.1%, compared to 95.8% in patients without such risk. In our analysis patients with GT3 achieved a lower SVR as compared to those infected with other genotypes (75.5%). However, it should be noted that our analysis involved all patients with HCV, regardless of therapeutic options, including a suboptimal regimen of SOF + RBV used predominantly in GT3-infected individuals, in contrast to the above-mentioned study, which focused solely on the new pangenotypic option—SOF/VEL. Two large analyses have also demonstrated that patients with GT3 infection and cirrhosis achieve lower SVR rates compared to patients with other genotypes [30, 31].

The effectiveness of various IFN-free therapeutic options was analyzed in a retrospective study conducted among 104 patients with PH (HVPG ≥ 6 mmHg), with 100 out of these patients achieving SVR, indicating a success rate of 96.2%. However, it is noteworthy that researchers did not report the effectiveness of treatment specifically within the subgroup of patients with CSPH concerning the presence of EV [27].

In a minor study that targeted HIV/HCV-coinfected patients with advanced liver fibrosis and PH (HVPG ≥ 6 mmHg), all subjects achieved SVR following treatment with IFN-free therapy based on SOF including 11 individuals diagnosed with CSPH (HVPG ≥ 10 mmHg) [28]. Our study, which included patients with HIV coinfection, demonstrated that 16 out of 19 (84.2%) individuals with EV achieved SVR. This compares to 55 out of 57 (96.5%) individuals without EV within the same subgroup.

In a large-scale RWE study, it was observed that DAA therapy was less effective in patients with EV [32]. Additionally, another study covering a patient population of nearly a thousand identified EV as an independent predictive factor for the lack of virological response to HCV treatment [33]. In our analysis, during the univariable analysis, factors such as male gender, GT3 infection, decompensation of liver function at baseline (B in the CP scale), and documented EV were identified as negative predictors of achieving SVR.

In our study, male gender appeared to be an independent predictor of failure of DAA therapy in patients with cirrhosis, confirming the results of other RWE analyses. One possible explanation for this phenomenon could have been poorer adherence in men compared to women, but this is only a hypothesis because in our study, which was retrospective, we did not assess adherence by objective methods. [34–36].

The DAA regimens evaluated in our study had a good safety profile, characterized by a low rate of treatment discontinuation due to adverse events, which is in line with clinical trials and other RWE studies [37, 38]. However, it is noteworthy, that the tolerability of DAA regimens was found to be less favorable in patients with EV.

Our analysis revealed a significantly higher prevalence of complications such as ascites, hepatic encephalopathy, and gastrointestinal bleeding in patients with EV compared to those without these complications. These findings are consistent with the natural progression of liver disease [39]. These observations suggest a need for heightened vigilance and closer monitoring of patients with EV undergoing DAA therapy, particularly given the increased risk of liver decompensation. Such patients should not be treated with regimens containing protease inhibitors (PI), according to current guidelines and the FDA (Food and Drug Administration) position, because of the potential risk of exacerbating liver dysfunction or liver failure [20, 40]. In our study, which has the character of a retrospective project from real-world clinical practice, the decision on the choice of therapy was entirely on the part of the treating physician, who in making it was guided by the provisions of the drug program and the current state of medical knowledge.

Although the first signals of the possibility of worsening liver function in patients with decompensated cirrhosis appeared in 2016, being the basis for a provision against their use in this population in the EASL recommendations 2016 published in 2017, the 2015 and all subsequent AASLD guidelines include the phrase “not recommended” for PI use in CP B and C patients [41–43]. Finally, the safety warning for PI-containing regimens was issued by the FDA in the second half of 2019, 107 patients with decompensated liver function at the very beginning of our study were treated with such regimens, mainly ombitasvir/ paritaprevir boosted with ritonavir ± dasabuvir ± RBV. Although 92 of them achieved SVR, 12 patients in this group discontinued treatment due to safety concerns related to deterioration of liver function. Six deaths were also reported, four of which were attributed to liver-related complications. Thus, our observations support recommendations suggesting that PI-containing regimens should be avoided in patients with decompensated cirrhosis qualified as CP class B or C. On the other hand, a large multicenter retrospective RWE study assessing the safety of 1,077 patients with advanced HCV cirrhosis, 42% of whom were treated with PI-containing regimens, showed no significant differences in safety compared to non-PI in those with MELD values up to 15 [44].

In the studied population, the prevalence of individuals classified as decompensated was significantly higher among patients with EV compared to those without varices, exceeding 25% as opposed to 6.8%, respectively. Notably, within the varices group, the SVR rate was below 90%. These findings resonate with those from another retrospective study, wherein patients with decompensated cirrhosis exhibited higher SVR rates in PP analysis, reaching 92.9% [38]. Patients with a low platelet count have an important impact on the patient’s prognosis in the course of cirrhosis [45]. In our analysis, such individuals had worse SVR rates in patients with EV than in those without EV.

Subgroups analyzed in our study demonstrated variances in the utilization of concomitant medications. Notably, within the EV group, patients were statistically more likely to receive beta-blockers aimed at reducing portal pressure and mitigating the risk of variceal bleeding. [46]. Additionally, there was an increased utilization of rifaximin within the EV subgroup, which corresponded to a higher baseline incidence of encephalopathy. It’s noteworthy that this medication not only addresses complications associated with liver cirrhosis but also potentially improves overall prognosis [47].

Diagnosis and treatment of HCV infection are of particular importance for this patient population, as effective treatment can favorably affect the progression of liver disease. The regression of liver fibrosis and CSPH following DAA treatment is of considerable interest but remains controversial. While some studies indicate rapid declines in liver stiffness measurement (LSM) and spleen stiffness measurement (SSM) following SVR, others suggest minimal changes [14, 48–50]. Conversely, improvements in MELD scores and liver function, along with reductions in HVPG, suggest the benefits of treatment in this population [49, 51].

As recommended by the European Association for the Study of the Liver (EASL), the accurate assessment of liver disease severity is crucial before initiating treatment for HCV infection, [20]. Specifically, individuals diagnosed with cirrhosis should undergo a comprehensive assessment to determine the presence of PH, which includes the evaluation for EV [20]. Additionally, individuals with EV identified during endoscopy before treatment should also undergo endoscopic examination to diagnose variceal recurrence after viral eradication. Traditionally, HVPG measurement has been considered the “gold standard” for assessing portal pressure. However, due to its invasiveness, there is growing interest in noninvasive methods to predict the presence of CSPH. Liver stiffness measurement has been identified as useful in assessing liver fibrosis [6]. Additionally, recent studies also show promising results for noninvasive techniques such as spleen stiffness measurement (SSM) in predicting CSPH. SSM, particularly using Acoustic Radiation Force Impulse (ARFI) imaging, has demonstrated a significant correlation between PH and the presence of EV [52, 53]. It is imperative to consider the long-term implications of HCV treatment, particularly in patients with PH. A previous report has shown that the risk of HCC development remains clinically significant in patients with PH complicated by advanced fibrosis even after successful eradication of HCV [16]. Variceal bleeding continues to be a serious problem despite advances in its treatment, with a mortality rate after a first bleeding episode as high as 15% [54].

Our study exhibits several limitations warranting attention. Firstly, our study did not employ HVPG measurements for the diagnosis of PH, relying instead on the presence of EV, which may not represent the gold standard in PH diagnosis. Another limitation is the potential interpersonal variability in diagnosing small esophageal varices. As this is a retrospective real-world study with patients from various hepatology centers, endoscopic examinations were performed by different specialists. Therefore, we lacked the option for an independent reviewer to verify these diagnoses and relied on existing medical documentation. Additionally, in this study we did not analyze all manifestations of PH, focusing mainly on EV. As a retrospective analysis of the real world, our study inevitably is burdened with inherent flaws, such as incomplete data, potential biases, data entry errors, data misclassification, and under-reporting of safety information, particularly the lack of detailed data on causes of death. In addition, the observational nature of the study may have introduced variability in treatment adherence, as the assessment of adherence was based solely on patient reports, with no objective method. The retrospective nature of the study also results in underestimation of adverse events, especially mild ones. Resistance-associated substitutions were not tested in this study. Patients with cirrhosis who did not undergo endoscopic evaluation were excluded from our analysis. Finally, our analysis lacked long-term data on liver function improvement following HCV eradication, as we did not have extended follow-up data on CP and MELD scores. Nevertheless, the strength of our investigation lies in the extensive analysis of a fairly large cohort of patients with EV across multiple hepatology centers, which may promote the generalization of the findings. Moreover, it is noteworthy that the percentage of patients lost to follow-up (3.5%) is relatively low, which is characteristic of controlled clinical trials rather than real-world studies.

## Conclusion

Our study highlights significant differences in treatment response and safety profile between patients with liver cirrhosis alone and those with liver cirrhosis and PH manifesting by EV. Patients with EV exhibited a lower SVR rate, indicating challenges in achieving optimal outcomes in this subgroup. Factors such as male gender, GT3 infection, EV, and higher CP scores were identified as negative predictors of SVR, emphasizing the need for personalized treatment approaches. The safety profile was notably worse in patients with EV, with a higher incidence of adverse events leading to treatment discontinuation, including serious liver-related complications. These findings underscore the importance of vigilant monitoring and timely intervention in patients with advanced liver disease.

## Data Availability

The datasets generated during and/or analysed during the current study are available from the corresponding author upon reasonable request.

## Abbreviations

CHC: Chronic hepatitis C
CSPH: Clinically significant portal hypertension
DAAs: Direct-acting antivirals
DSV: Dasabuvir
EASL: European Association for the Study of the Liver
EV: Esophageal varices
FDA: Food and Drug Administration
GT: Genotype
HBV: Hepatitis B virus
HCC: Hepatocellular carcinoma
HCV: Hepatitis C virus
HIV: Human immunodeficiency virus
HVPG: Hepatic venous pressure gradient
IFN: Interferon
INR: International normalized ratio
LSM: Liver stiffness measurement
MELD: Model for end-stage liver disease
NHF: National Health Fund
OBV: Ombitasvir
OR: Odds ratio
PH: Portal hypertension
PI: Protease inhibitors
PP: Per protocol
PTV/r: Paritaprevir boosted with ritonavir
RBV: Ribavirin
RNA: Ribonucleic acid
RWE: Real-world evidence
SAE: Severe adverse events
SOF: Sofosbuvir
SSM: Spleen stiffness measurement
SVR: Sustained virological response
VEL: Velpatasvir
